# Implications of Kenya's Enhanced Public Officers Medical Scheme on Equity in Strategic Purchasing and Progress Towards Universal Health Coverage

**DOI:** 10.1002/puh2.70418

**Published:** 2026-10-08

**Authors:** David Olpengs, Diana Moraa, Boniface Mbuthia

**Affiliations:** ^1^ Health Systems Strengthening Directorate Amref Health Africa Nairobi Kenya; ^2^ Health Economics Research Unit KEMRI‐Wellcome Trust Nairobi Kenya

**Keywords:** equity, Public Officers Medical Scheme Fund (POMSF), Social Health Authority (SHA), social health insurance act, strategic health purchasing, universal health coverage (UHC)

## Abstract

**Background:**

Kenya transitioned its health financing from the National Health Insurance Fund (NHIF) to the Social Health Authority (SHA) in 2024. This model was designed to advance efficiency and equity, ultimately leading to universal health coverage (UHC). Shortly thereafter, the government introduced a parallel Enhanced Public Officers Medical Scheme Fund (POMSF), tailored to privilege public officers through dedicated funding and benefits. This article explores the implications of this targeted scheme on equity in strategic purchasing and the journey towards UHC.

**Methodology:**

The article is a policy perspective grounded on document review of legislation, regulations, government documents and grey literature. Evidence was identified through targeted searches of PubMed, Google Scholar and Kenyan sources. Analysis was guided by the Strategic Purchasing Resource Centre for Africa (SPARC) and RESYST strategic purchasing frameworks and organized across governance, revenue mobilization, risk pooling, benefit design, provider contracting, provider payment, access protocols, financial protection and accountability. Comparative evidence from civil‐servant and segmented insurance arrangements in Thailand, Tanzania and Indonesia was used.

**Findings:**

SHA is designed to support population coverage, cross‐subsidization, standardized entitlements and primary healthcare–led access. POMSF, although administered through SHA, relies on ring‐fenced public financing, a scheme‐specific governance structure, enhanced benefits, separate contractual arrangements, negotiated tariffs and flexible access pathways for public officers. These features may improve financial protection and service responsiveness for beneficiaries but risk‐fragmenting pools, weakening redistribution, creating unequal entitlements and generating competing provider incentives.

**Conclusions:**

POMSF is not inherently incompatible with UHC, but its equity effects depend on how it is financed, governed and linked to SHA. Kenya should establish transparent financial links, common purchasing standards, harmonized tariffs and referral rules, integrated data and accountability systems, and periodic equity review. Without these safeguards, POMSF may entrench a two‐tier system, weaken solidarity within health financing and Kenya's movement towards equitable UHC.

## 1 Introduction

In October 2023, Kenya enacted and assented the Social Health Insurance Act, 2023, with the Act officially commencing on 22 November 2023 and designating 1 October 2024 as the ‘appointed day’ for transition from the National Hospital Insurance Fund (NHIF) to the new Social Health Authority (SHA) model aimed at advancing a unified approach emphasizing equity, efficiency and universal health coverage (UHC) [1, 2]. However, within weeks of the October 2024 roll‐out, the government established a separate Public Officers Medical Scheme via the Public Finance Management (Public Officers Medical Scheme Fund) Regulations, 2024 [3]. Notably, Clause 31(3) of the Social Health Insurance Bill, 2023, had expressly authorized that ‘The Authority may, subject to such guidelines as it may issue, provide an enhanced benefit package for civil servants and retired civil servants’ (1), but this provision was omitted from the enacted Act [4]. The enhanced‐cover principle was subsequently reintroduced for public officers through Regulation 14(2)(c) of the Public Finance Management (Public Officers Medical Scheme Fund) Regulations, 2024, which requires the scheme contract to provide ‘a benefits package equivalent or higher to the Comprehensive Medical Insurance Scheme for Civil Servants’ available immediately before the Regulations commenced (3). Although the SHA reforms aim to promote UHC and equity, the creation of a privileged fund for public officers introduces both opportunities and significant risks for equity and UHC.

Global experience illustrates both the risks of segmentation and possible reform pathways. Thailand maintains a tax‐financed Civil Servant Medical Benefit Scheme (CSMBS) alongside the Universal Coverage Scheme (UCS). Although both provide broad coverage, the CSMBS has historically offered more flexible access and used fee‐for‐service for outpatient care, contributing to substantially higher per‐capita expenditure and weaker expenditure control than the capitation‐ and global‐budget‐based UCS [5, 6]. Tanzania has similarly operated differentiated arrangements, with the National Health Insurance Fund initially focused on public employees and Community Health Funds serving largely informal and rural populations; the resulting fragmentation has constrained redistribution and complicated coherent purchasing [7]. Indonesia followed a different path by consolidating Askes for civil servants and other employment‐ and poverty‐targeted schemes into the national Jaminan Kesehatan Nasional programme, illustrating how de‐fragmentation can establish a common purchaser and entitlement framework, even though implementation and equity challenges remain [8, 9].

These experiences suggest that a civil‐servant scheme is not inherently incompatible with UHC, but its effects depend on how it relates to the national pool and purchasing system. Using a structured assessment criterion drawn from Strategic Purchasing Resource Centre for Africa (SPARC) and RESYST purchasing progress tracking frameworks [10, 11], this article critically examines the implications of this enhanced scheme on equity in strategic purchasing and progress towards UHC.

## 2 Methodology

This article is a policy perspective that is grounded on a targeted document review of legislative instruments, government policy documents and published and grey literature examining Kenya's recent health financing reforms. Searches were undertaken in PubMed, Google Scholar and official government portals, including Kenya Law, the Ministry of Health and SHA websites, using terms related to the SHA, the Public Officers Medical Scheme Fund (POMSF) and strategic purchasing in Kenya. Documents published between 2020 and 2025 that directly addressed SHA reforms or the design and operation of POMSF were eligible for inclusion. In total, 53 records were identified, of which 19 met the inclusion criteria following title, abstract and full‐text screening. Data were extracted using structured matrices aligned to the SPARC strategic purchasing framework and synthesized through comparative analysis of SHA and POMSF to identify points of convergence, divergence and their implications for equity in strategic purchasing and progress towards UHC. Evidence was triangulated to identify consistent patterns across five core thematic dimensions: revenue mobilization, risk pooling, benefit package design, provider payment and contracting, and access protocols.

## 3 Findings and Discussion

### 3.1 Governance and Oversight

Under the general SHA reforms, the SHA Board comprises 11 members, including the MoH, Treasury, Council of Governors, professional associations, labour unions and, crucially, a nominee of the informal sector ensuring that voices of historically underserved groups are heard in policy design [1, 4]. Regulation 10(1) of the Public Finance Management (Public Officers Medical Scheme Fund) Regulations, 2024 assigns oversight of POMSF to the SHA Board, whereas Regulation 11(1) establishes an advisory Committee comprising four representatives of the Ministry responsible for public service, four SHA representatives, one representative of the Ministry responsible for finance, one representative of the Office of the Attorney‐General and two nominees of the Union of Kenya Civil Servants [3]. Although this structure ensures focused oversight for civil servants and may improve responsiveness and accountability, its composition and scheme‐specific mandate create a plausible governance risk that public officers’ preferences may receive more organized and sustained attention than equity concerns affecting the broader SHA population, potentially skewing policy towards better resourced groups.

### 3.2 Revenue Mobilization

Under the general SHA model, salaried workers contribute 2.75% of their gross monthly income, whereas unsalaried participants undergo Proxy Means Testing (PMT) to determine annual premiums, with subsidies for indigents [1, 4]. Public officers are not exempt from this contribution; they also pay 2.75% of their gross income into the Social Health Insurance Fund (SHIF), whereas POMSF draws from parliamentary appropriations and the health medical benefits of public officers [3]. Accordingly, POMSF does not absolve public officers from the mandatory SHIF contribution; rather, it may reduce or eliminate additional out‐of‐pocket payments at the point of service for benefits covered by the enhanced scheme. This financing arrangement nevertheless creates an embedded fiscal inequity where public funds are preferentially allocated towards a minority group already advantaged within the labour market. In addition, those public resources are ring‐fenced away from broader UHC financing priorities.

### 3.3 Resource Pooling and Risk Sharing

The structure of SHA is intended to facilitate broad risk pooling and cross‐subsidization across income levels, health statuses and age groups. By enrolling the entire population into its three funds, the system aims to redistribute resources between the wealthy and the poor, from the healthy to the sick, and from the young to the old [1]. The establishment of POMSF as a separate fund for public officers, however, introduces an additional pooling arrangement alongside SHA. By isolating a relatively well‐off and formally employed segment of the population from the national risk pool with ring‐fenced resources and enhanced benefits, POMSF weakens the extent to which public financing contributes to cross‐subsidization within the wider system. Although the scheme has not yet been formally evaluated, this introduces the risk of undermining equity goals and may lead to the perpetuation of two‐tier coverage, where public servants benefit from more secure and potentially better resourced services than the general population. Such an outcome would be inconsistent with the solidarity principle that financing arrangements should distribute resources and access fairly across population groups.

### 3.4 Benefits Package Design

The initially fragmented health benefit packages in NHIF were consolidated under SHA to offer a standard, comprehensive package, including outpatient PHC, emergency treatment, inpatient care, cancer screening, assistive devices and palliative services which are subject to conditional limits on some benefits [1, 12]. The POMSF guarantees an enhanced, much more generous package equal to or exceeding the former Comprehensive Medical Insurance Scheme for Civil Servants, with no waiting periods or conditional exclusions cited [3]. This effectively preserves public officers’ entitlements. However, differentiated entitlements may institutionalize unequal access, breaching UHC's universality principle and undermining strategic purchasing efforts to align resources with population health needs by creating a two‐tier entitlement structure favouring a narrow group in the population.

### 3.5 Provider Contracting: Empanelment

The empanelment of both public and private facilities by SHA is under uniform standards as provided by the Kenya Medical Practitioners and Dentists Council (KMPDC) [1, 4]. The POMSF utilizes SHA's contracting model but may negotiate bespoke service‐level agreements or preferential provider lists on behalf of civil servants [3]. The POMSF Regulations require participating public employers to enter into annual contracts with SHA, authorize the Scheme Committee to review the list of contracted healthcare providers and allow each contract to specify the benefits package and access rules [3]. In implementation, SHA signed POMSF‐specific contracts in June 2026 with private and faith‐based Level 3–6 facilities and separately tendered a 3‐year framework agreement for emergency air‐evacuation services exclusively for POMSF beneficiaries [13, 14]. Although these tailored contracts may improve service standards and accountability, the selective empanelment has the risk of attracting top‐tier providers towards POMSF beneficiaries, widening inequities in access including public–private disparities.

### 3.6 Provider Payment Mechanisms (PPMs)

Under SHA, payment methods are designed to be standardized and cost‐controlled across all providers, which include capitation (global budgets at primary care level), case‐based bundles, fee‐for‐service and per diems for SHIF and ECCIF [1, 4]. POMSF now operates through a separately negotiated reimbursement schedule rather than only a theoretical power to vary payment terms. In April 2026, a government‐led joint communiqué suspended strict application of the initial POMSF tariff clause pending nationwide negotiations; in June 2026, SHA and private and faith‐based Level 3–6 providers signed contracts incorporating negotiated POMSF tariffs and terms for service delivery, reimbursement and accountability [13, 15]. Although these negotiated reimbursements may improve predictability, provider participation and service quality for public officers, the equity concern is ring‐fenced financing, and separately negotiated terms may offer more attractive or predictable reimbursement, potentially encouraging providers to prioritize POMSF beneficiaries. Publication of comparative tariffs and claim‐settlement times is needed to test this effect.

### 3.7 Access Protocols

SHA enforces gatekeeping protocols through Primary Care Networks (PCNs) to regulate access to higher level facilities [4, 12]. However, the implementation of this model is constrained by limited PHC capacity, with only 7% service readiness reported nationally as of 2023 [16]. Public officers face no formal gatekeeping restrictions under POMSF [3]; rather they have a more flexible and often direct access to specialists and higher level hospitals. Although this increases convenience and timely specialist care for public officers, it weakens the national push for PHC‐led access as the cornerstone for UHC. Whether these differing access protocols translate into stratified care pathways will depend on beneficiary and provider responses and should be assessed empirically as implementation progresses.

Table 1 summarizes the principal differences between SHA and POMSF across the key strategic purchasing domains and outlines their potential implications for equity and progress towards UHC.

**TABLE 1:** Comparison of Social Health Authority (SHA) and Public Officers Medical Scheme Fund (POMSF) across key strategic purchasing domains.

Domain	Social Health Authority (SHA)	Public Officers Medical Scheme Fund (POMSF)	Implications for equity and strategic purchasing
Governance and oversight	Governed by the SHA Board, which includes representation from national and county governments, professional bodies, labour organizations and the informal sector	Overseen by the SHA Board, with an additional scheme‐specific advisory committee comprising representatives of public service institutions, SHA, the National Treasury, the Attorney‐General and civil servants’ unions	Dedicated representation can strengthen responsiveness to public officers but may give their interests greater institutional influence than those of less‐organized population groups
Revenue mobilization	Salaried members contribute 2.75% of gross income, whereas contributions for non‐salaried households are determined through means assessment, with government subsidies intended for indigent households	Public officers make the mandatory SHA contribution, whereas enhanced benefits are financed through parliamentary appropriations and medical‐benefit allocations from participating public employers	Additional public funding for a relatively advantaged employment group may create fiscal inequity where comparable resources are unavailable for the wider population
Risk pooling and cross‐subsidization	Intended to support broad pooling and redistribution across income, health‐risk and demographic groups	Operates through ring‐fenced resources for public officers alongside the wider SHA financing structure	Separate funding limits the extent to which additional public resources support redistribution across the national population and may weaken solidarity within the health financing system
Benefits package	Provides a standardized national package covering primary, emergency, inpatient, chronic and other specified services, subject to defined tariffs, conditions and limits	Required to provide a package equivalent to or better than the former Comprehensive Medical Insurance Scheme for Civil Servants	Unequal benefit entitlements may create a two‐tier system in which access is determined partly by employment status rather than health need
Provider contracting and empanelment	Contracts eligible public, private and faith‐based providers under national empanelment and regulatory requirements	Uses SHA as the purchasing agency but allows scheme‐specific contracts, provider arrangements and services, including POMSF‐specific contracting with private and faith‐based facilities	Separate provider arrangements may attract providers and service capacity toward POMSF beneficiaries, potentially reducing equitable access for the general population
Provider payment mechanisms	Uses standardized payment approaches, including capitation or global budgets at primary care level, case‐based payments, fee‐for‐service and per diem arrangements	Uses separately negotiated tariffs and contractual terms for service delivery, reimbursement and accountability	Where POMSF payment terms are more attractive or predictable, providers may prioritize its beneficiaries. Comparative publication of tariffs and claim‐settlement periods is required to establish whether this occurs
Access and referral protocols	Promotes primary healthcare–led access and gatekeeping through Primary Care Networks, although implementation is constrained by limited facility readiness	Allows more flexible access arrangements that may include the direct use of specialists and higher level facilities, depending on the scheme contract	Different access rules may weaken PHC‐led gatekeeping and produce stratified care pathways, with public officers obtaining faster or more direct access to higher level services

*Source:* Authors’ synthesis based on the Social Health Insurance Act and Regulations, and the Public Finance Management (Public Officers Medical Scheme Fund) Regulations.

## 4 Policy Recommendations

Kenya has four broad policy options. First, POMSF could be fully integrated into SHA, with public officers receiving the same national package and any employment benefit provided through non‐clinical allowances or regulated supplementary insurance. Second, POMSF could remain as a supplementary fund but only after public officers’ contributions and a defined public subsidy are paid into the common SHA pool. Third, the two arrangements could remain legally distinct while using common benefits, tariffs, referral rules, provider contracts, data systems and accountability standards. Fourth, the status quo could continue with separate benefits and purchasing rules, but this option carries the greatest risk of inequity and incoherent provider incentives. The global examples favour consolidation where feasible and harmonization and where political or institutional constraints prevent immediate merger [17, 18, 19]. Indonesia demonstrates the value of bringing civil‐servant and other schemes under a common national architecture, whereas Thailand shows the persistent expenditure and equity consequences that can arise when a protected civil‐servant scheme retains distinct access and payment arrangements [5, 8].

## 5 Conclusion

Kenya's UHC transition includes whether purchasing arrangements allocate public resources and access according to health need. The equity effect of POMSF therefore depends less on the existence of additional benefits for public officers than on how those benefits are financed, contracted and governed. Transparent financial links between the schemes, a common floor for benefits and purchasing rules, and joint public reporting of allocations, payment performance, service access and outcomes across beneficiary groups would allow the scheme to meet legitimate workforce‐protection objectives without diverting fiscal and provider capacity from the wider population. Without these safeguards, the institutional precedent of privileged parallel coverage may prove more difficult to reverse than the immediate financing imbalance. The policy priority is therefore to make POMSF explicitly accountable to national UHC objectives through a sequenced harmonization process and mandatory review.

## Author Contributions

**Boniface Mbuthia**: writing – review and editing, validation. **David Olpengs**: conceptualization, methodology, writing – original draft, writing – review and editing. **Diana Moraa**: writing – review and editing.

## Funding

The authors have nothing to report.

## Ethics Statement

The authors have nothing to report.

## Consent

The authors have nothing to report.

## Conflicts of Interest

The authors declare no conflicts of interest.

## Data Availability

Data sharing is not applicable to this article as no datasets were generated or analysed during the current study.

## References

[1] The Kenya Law, The Social Health Insurance Act No. 16 of 2023, Kenya Gazette Vol. CXXV No. 249 (2023), https://new.kenyalaw.org/akn/ke/act/2023/16/eng@2023‐11‐24.

[2] Health Intervention and Technology Assessment Program (HITAP) , National Hospital Insurance Fund (NHIF) Health Financing Reforms Experts’ Panel (HEFREP) Visit to Thailand on Universal Health Coverage (UHC) and Health Technology Assessment (HTA) June 5th–7th, 2019 (2019). https://www.hitap.net/en/document/nhif‐hefrep‐uhc‐hta‐visit‐thailand/.

[3] The Kenya Law , The Public Finance Management (Public Officers Medical Scheme Fund) Regulations, Kenya Gazette Vol. CXXVI No. 230 (2024), https://new.kenyalaw.org/akn/ke/act/ln/2024/195/eng@2024‐12‐27.

[4] The Kenya Law, The Social Health Insurance Regulations, Legal Notice 49 of 2024 . (2024), https://new.kenyalaw.org/akn/ke/act/ln/2024/49/eng@2024‐03‐08.

[5] W. Patcharanarumol , W. Panichkriangkrai , A. Sommanuttaweechai , K. Hanson , Y. Wanwong , and V. Tangcharoensathien , “Strategic Purchasing and Health System Efficiency: A Comparison of Two Financing Schemes in Thailand,” PLoS ONE 13, no. 4 (2018): e0195179, 10.1371/journal.pone.0195179. PMC5880375 29608610

[6] V. Tangcharoensathien , S. Limwattananon , W. Patcharanarumol , J. Thammatacharee , P. Jongudomsuk , and S. Sirilak , “Achieving Universal Health Coverage Goals in Thailand: The Vital Role of Strategic Purchasing,” Health Policy and Planning 30, no. 9 (2015): 1152–1161, 10.1093/heapol/czu120. PMC4597041 25378527

[7] S. Renggli , I. Mayumana , C. Mshana , et al., “Looking at the Bigger Picture: How the Wider Health Financing Context Affects the Implementation of the Tanzanian Community Health Funds,” Health Policy and Planning 34, no. 1 (2019): 12–23, 10.1093/heapol/czy091. PMC6479827 30689879

[8] R. Agustina , T. Dartanto , R. Sitompul , et al., “Universal Health Coverage in Indonesia: Concept, Progress, and Challenges,” Lancet 393, no. 10166 (2019): 75–102, 10.1016/S0140-6736(18)31647-7. 30579611

[9] N. Mboi , “Indonesia: On the Way to Universal Health Care,” Health Systems & Reform 1, no. 2 (2015): 91–97, 10.1080/23288604.2015.1020642. 31546309

[10] RESYST , What is Strategic Purchasing for Health? [Internet] (RESYST, 2014), https://resyst.lshtm.ac.uk/sites/resyst/files/content/attachments/2018‐08‐22/What%20is%20strategic%20purchasing%20for%20health.pdf.

[11] A. Gatome‐Munyua , I. Sieleunou , E. Barasa , et al., “Applying the Strategic Health Purchasing Progress Tracking Framework: Lessons From Nine African Countries,” Health Systems & Reform 8, no. 2 (2022): e2051796, 10.1080/23288604.2022.2051796. PMC7613345 35446229

[12] Ministry of Health, Kenya Tariffs To The Benefit Package Under The Social Health Insurance Act No. 16 of 2023 (2023) , https://health.go.ke/sites/default/files/2024‐11/TARIFFS%20TO%20THE%20BENEFIT%20PACKAGE%20TO%20THE%20SHI.pdf.

[13] Ministry of Health , Government Signs POMSF Contracts, Launches SHA Biometric Registration for Dependents (Ministry of Health, 2026), https://www.health.go.ke/government‐signs‐pomsf‐contracts‐launches‐sha‐biometric‐registration‐dependents.

[14] The Social Health Authority, Framework Agreement for the Provision of Emergency Air Evacuation Services for Beneficiaries of the Public Officers Medical Scheme Fund for a Period of Three (3) Years , (2026) https://tenders.go.ke/storage/Documents/1772531728597‐tender‐document.pdf.

[15] Ministry of Health , CS Duale Chairs Signing of Joint Communiqué to Streamline POMSF Service Delivery (Ministry of Health, 2026), https://www.health.go.ke/cs‐duale‐chairs‐signing‐joint‐communique‐streamline‐pomsf‐service‐delivery?.

[16] Ministry of Health, Kenya Health Facility Census Report 2023 (Ministry of Health, 2023), https://www.health.go.ke/sites/default/files/2024‐01/Kenya%20Health%20Facility%20Census%20Report%20September%202023.pdf.

[17] I. Mathauer , P. Saksena , and J. Kutzin , “Pooling Arrangements in Health Financing Systems: A Proposed Classification,” International Journal for Equity in Health 18, no. 1 (2019): 198, 10.1186/s12939-019-1088-x. PMC6925450 31864355

[18] I. Mathauer , L. Vinyals Torres , J. Kutzin , M. Jakab , and K. Hanson , “Pooling Financial Resources for Universal Health Coverage: Options for Reform,” Bulletin of the World Health Organization 98, no. 2 (2020): 132–139, 10.2471/BLT.19.234153. PMC6986215 32015584

[19] E. Barasa , I. Mathauer , E. Kabia , et al., “How Do Healthcare Providers Respond to Multiple Funding Flows? A Conceptual Framework and Options to Align Them,” Health Policy and Planning 36, no. 6 (2021): 861–868, 10.1093/heapol/czab003. PMC8227448 33948635

